# Study on Spatial and Temporal Characteristics of Surface Albedo at the Northern Edge of the Badain Jaran Desert Based on C + STNLFFM Model

**DOI:** 10.3390/s22176494

**Published:** 2022-08-29

**Authors:** Peng He, Rutian Bi, Lishuai Xu, Fan Yang, Jingshu Wang, Chenbin Cao

**Affiliations:** 1 College of Resources and Environment, Shanxi Agricultural University, Jinzhong 030801, China; 2 Instituste of Desert Meteorology, China Meteorological Administration, Urumqi 830002, China

**Keywords:** Badain Jaran Desert, surface albedo, C model, STNLFFM model, geographical detector model

## Abstract

Obtaining surface albedo data with high spatial and temporal resolution is essential for measuring the factors, effects, and change mechanisms of regional land-atmosphere interactions in deserts. In order to obtain surface albedo data with higher accuracy and better applicability in deserts, we used MODIS and OLI as data sources, and calculated the daily surface albedo data, with a spatial resolution of 30 m, of Guaizi Lake at the northern edge of the Badain Jaran Desert in 2016, using the Spatial and Temporal Non-Local Filter-based Fusion Model (STNLFFM) and topographical correction model (C model). We then compared the results of STNLFFM and C + STNLFFM for fusion accuracy, and for spatial and temporal distribution differences in surface albedo over different underlying surfaces. The results indicated that, compared with STNLFFM surface albedo and MODIS surface albedo, the relative error of C + STNLFFM surface albedo decreased by 2.34% and 3.57%, respectively. C + STNLFFM can improve poor applicability of MODIS in winter, and better responds to the changes in the measured value over a short time range. After the correction of the C model, the spatial difference in surface albedo over different underlying surfaces was enhanced, and the spatial differences in surface albedo between shifting dunes and semi-shifting dunes, fixed dunes and saline-alkali land, and the Gobi and saline-alkali land were significant. C + STNLFFM maintained the spatial and temporal distribution characteristics of STNLFFM surface albedo, but the increase in regional aerosol concentration and thickness caused by frequent dust storms weakened the spatial difference in surface albedo over different underlying surfaces in March, which led to the overcorrection of the C model.

## 1. Introduction

The Badain Jaran Desert is located in the center of the Alxa Plateau, China; it lies at the end of the southeast monsoon in the East Asian monsoon system [1]. This desert’s special geographical location is distinct from those of tropical, subtropical, and coastal deserts, which are controlled by subtropical high-pressure systems or cold ocean currents [2]. Ground-air interactions in the Badain Jaran Desert significantly affect extreme weather events in China and Asia, and even influence global climatological and ecological changes [3]. Surface albedo refers to the ratio of total solar radiation reflected from the surface to the total incident solar radiation energy; it indicates the reflectivity of the surface to solar radiation. Surface albedo is an important parameter in land surface process and climate models [4,5]. Slight changes in surface albedo can significantly affect the radiation energy balance of the ground-air system, resulting in local, regional, and even global climate change. Particularly in desert areas, surface albedo has a remarkable effect on the regional energy balance and water-heat exchange [6]. Deserts have low vegetation cover due to their scarce precipitation levels, which mean that they have a relatively high surface albedo. Thus, the net radiation obtained at the surface is relatively low, and consequently both the sensible and latent heat fluxes are also low, resulting in weakened atmospheric convergence and reduced clouds and precipitation. This causes persistent drought in desert areas, while enhancing and expanding further desertification [7,8]. Therefore, accurately calculating surface albedo is greatly important when studying ground-air interactions in regions such as the Badain Jaran Desert.

However, parameterization schemes are still widely used to calculate surface albedo in climate studies and land surface simulations; this results in poor accuracy regarding regional-scale albedo [9]. Compared with ground-based experimental observations, remote sensing can quickly determine the surface albedo of continuously distributed regions [10]. There are a range of diverse products that can simulate surface albedo, specifically ground-atmosphere exchange, including the Advanced Very High-Resolution Radiometer (AVHRR), the Moderate Resolution Imaging Spectroradiometer (MODIS), and the Multi-angle Imaging SpectroRadiometer (MISR). In the absence of high-resolution data, the differences between low-resolution data and ground measurements can complicate the in-depth investigation of the ground-atmosphere exchange process [11,12]. To solve this problem, Cherechali et al. [13] used the least-squares method, as well as a linear spectral hybrid model to transform low-spatial-resolution images into high-spatial-resolution images. Liang et al. [14,15] proposed a method for calculating surface albedo via the conversion of different narrowband sensors (e.g., Thermic Mapper (TM), Enhanced Thematic Mapper Plus (ETM+), and Operational Land Imager (OLI)) to broadband sensors. Shuai et al. [16] converted Landsat spectral albedo data to surface broadband albedo data, basing the conversion on the surface bidirectional reflectance distribution function (BRDF) obtained by MODIS. He et al. [17] proposed a unified algorithm that can perform a radiation transfer simulation on the Landsat spectral response function and the BRDF database. Based on atmospheric apparent reflectance data acquired by the Multispectral Scanner System (MSS), TM, ETM+, and OLI, this algorithm can directly estimate surface albedo using few auxiliary input data points. However, limitations in temporal resolution mean that surface albedo calculations based on high-resolution data inversion are not sufficient for studying ground-atmosphere exchange processes in desert areas [18]. The spatiotemporal fusion of multi-source remote sensing data offers an effective solution to this dilemma [19,20]. These methods can overcome the performance constraints of a single sensor, and provide complementary observations from multiple platforms, thus attaining more accurate and comprehensive process monitoring [21]. Spatiotemporal data fusion algorithms can be classified into three types, depending on whether they are based on pixel unmixing [22,23], spatiotemporal filtering [24,25,26], or sparse expression [27,28]. Data fusion methods based on spatiotemporal filtering are widely used because of their advantages (e.g., simple framework, ease of use, and high prediction accuracy) [29]. Compared with other algorithms, the Spatial and Temporal Non-Local Filter-based Fusion Model (STNLFFM) algorithm can predict remote sensing image sequences based on a high spatiotemporal redundancy. Moreover, it can improve fusion accuracy during the conversion of multi-temporal low-resolution and multi-temporal high-resolution data [26,30].

At present, STNLFFM has been widely used in building vegetation indexes, building indexes, and water indexes, calculated by using reflectance data [19,21]. Due to the spatial heterogeneity and complexity of land cover, spatiotemporal data fusion is still rarely used in the study of surface albedo [31]. Rugged terrain, as a spatial heterogeneity factor, affects the estimation of surface albedo in high- and low-resolution data. For the high-resolution data, the surface albedo is mainly affected by the slope, the aspect of the single slope, and the surrounding terrain [31]. The topographic effects in low resolution data are often overlooked because the overall slope of coarse-resolution pixels is usually small, and the adjacent reflectance is negligible. In practice, the topographic variation within one pixel is significant for low-resolution data in rugged terrains, and is treated as a modification to a BRDF due to the shadows and the tilt on a microarea surface within one pixel [32]. Simply neglecting the topographic effects in surface albedo modeling and retrievals can lead to large biases and uncertainties. Topographical correction is necessary for the spatiotemporal fusion algorithms of surface albedo.

Based on MODIS and OLI data, and using the STNLFFM combined with a topographical correction model (the C correction model), we acquired surface albedo data for Guaizi Lake at the northern edge of the Badain Jaran Desert. We acquired these data daily, at a spatial resolution of 30 m. We compared the accuracy and spatiotemporal characteristics of surface albedo obtained using STNLFFM and C + STNLFFM, with the aim of obtaining surface albedo data with improved accuracy and applicability in desert areas.

## 2. Materials and Methodology

### 2.1. Study Area

**Figure 1:**
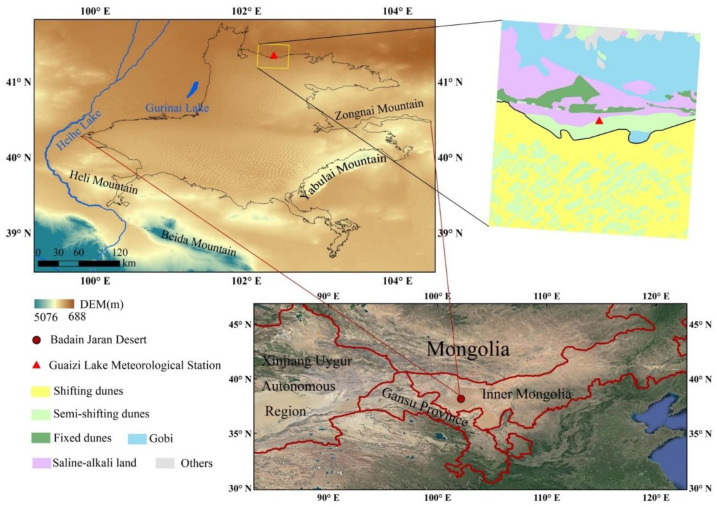
The location of meteorological station and different underlying surfaces of the study area. DEM and the study area within the China map are from the Geospatial Data Cloud (http://www.gscloud.cn (accessed on 5 April 2021)) and Google Earth, respectively.

The Badain Jaran Desert is located at the border of the Inner Mongolia Autonomous Region and Gansu Province in northwestern China. It is bounded to the east by Zongnai Mountain, to the west by the Heihe River, to the north by Guanzi Lake, and to the south by Heli Mountain, Beida Mountain, and Yabulai Mountain (Figure 1). The desert covers a total area of 5.2 × 10^4^ km^2^; it is the second largest flowing desert in China. The Badain Jaran Desert has an undulating topography, with high terrain in the southeast, and low terrain in the northwest. Specifically, high, dense sand dunes are distributed in the southeast, with the topography being flat in the west (adjacent to the Heihe River basin). The topography is the lowest in the north, with a great degree of undulation [33]. The Guaizi Lake Meteorological Station is located at the northern edge of the Badain Jaran Desert; this area has a typical inland temperate desert climate, with westerly winds prevailing all year round. Specifically, the average annual wind speed is 4.96 m·s^−1^, with an average of 62.1 and 28.9 windy and sand storm days per year, respectively; they mostly occur in spring [34]. The region is droughty, with little rainfall all year round. The average annual precipitation is only 42.9 mm, with the potential evaporation being nearly 100 times higher than precipitation. Moreover, the temperature difference between summer and winter is very significant [35]. The influence of local topography (e.g., river valleys and mountain ranges) means that there are a diverse range of underlying surfaces around the meteorological station (including shifting dunes, semi-shifting dunes, fixed dunes, the Gobi, saline-alkali land, and others).

### 2.2. Data

We acquired MODIS albedo data from the MCD43A1, MCD43A2, and MCD43A3 instruments (https://ladsweb.modaps.eosdis.nasa.gov (accessed on 10 May 2021)) with a spatial resolution of 500 m. MCD43A2 can determine the local noon solar zenith angle, which allowed for the sky scattering factor to be calculated accordingly. Here, we selected black sky shortwave albedo and white sky shortwave albedo from the MCD43A33 albedo data; we calculated the MODIS-based real surface albedo according to the sky scattering factor.

We downloaded a total of 32 OLI images from 2016 from the Geospatial Data Cloud (http://www.gscloud.cn (accessed on 5 April 2021)); the orbit numbers and acquisition times are described in Table 1. We calculated the surface reflectance through radiometric calibration and Fast Line-of-sight Atmospheric Analysis of Spectral Hypercubes (FLAASH) with atmospheric corrections. We calculated the Albedo-to-Nadir (AN) coefficient according to high-quality (QA = 0) MODIS BRDF data from MCD43A1; we determined the OLI spectral albedo by multiplying the AN coefficient with the surface albedo. Finally, we calculated the OLI surface short-wave broad-band albedo according to the OLI narrow-band to broad-band conversion coefficient (as described in Equation (1)):

(1) $$\alpha_{\mathrm{short}}=0.356\times \alpha_{2}+0.13\times \alpha_{4}+0.373\times \alpha_{5}+0.085\times \alpha_{6}+0.072\times \alpha_{7}-0.0018$$

where, α_short_ denotes the OLI surface shortwave broadband albedo, and α_2_, α_4_, α_5_, α_6_, and α_7_ denote the surface feature spectral reflectance data of blue, red, near-red, and two mid-red bands in the OLI data, respectively (the central wavelengths of these two bands are 1.560–1.651 and 2.100–2.300 µm, respectively).

We obtained desert type data from the *1:100,000 Distribution Atlas of Chinese Deserts* provided by the Environmental and Ecological Science Data Center for Western China (http://westdc.westgis.ac.cn (accessed on 1 January 2021)). According to this atlas, the main underlying surface types near the Guaizi Lake meteorological station include shifting dunes, semi-shifting dunes, fixed dunes, the Gobi, and saline-alkali land. In addition, we generated an Advanced Spaceborne Thermal Emission and Reflection Radiometer (ASTER) global digital elevation model (GDEM; 30 m) of the corresponding scale range from the Geospatial Data Cloud, using daily wind speed and surface albedo measurements from 2016 recorded at the Guaizi Lake meteorological station.

### 2.3. Analysis Method

#### 2.3.1. C Correction Model

The C correction model assumes that there is a linear relationship between the imaging radiation value and the cosine of the incident angle (Equation (2)). The correction coefficient, C, can be introduced into the cosine correction model to address over-corrections arising from the adjustment of the scattered radiation of the sky, and the reflected radiation of the neighboring terrain [36]. In Equation (2), ρ_s_ denotes the uncorrected reflectance before correction, *i_t_* denotes the incident angle of sunlight, and cosi_t_ denotes the illumination coefficient, which is calculated as shown in Equation (3). In Equation (3), θ_s_ denotes the solar zenith angle, σ denotes the angle of the slope, *β* denotes the solar azimuth angle, ω denotes the angle of the aspect, and C denotes the ratio of the intercept b to the slope a regarding the linear regression equation between ρ_s_ and the illumination coefficient cosi_t_ (Equation (4)).

(2) $$\rho_{s}=a\times {\cos i}_{t}+b$$

(3) $${\cos i}_{t}=\cos(\sigma )\times {\;\cos(\theta }_{s})\;+\;\sin(\sigma )\times {\sin(\theta }_{s})\times \;\cos(\beta -\omega )$$

(4) C=b/a

The corrected surface reflectance, ρ_c_, is determined by substituting C into Equation (5).

(5) $$\rho_{c}{=\rho }_{s}\times \frac{{\cos\theta }_{s}+C}{{\cos i}_{t}+C}$$

#### 2.3.2. STNLFFM

STNLFFM makes full use of two or more known temporal high- and low-resolution image pairs, as well as known low-resolution images at the predicted moment, to predict the high-resolution fused images at the corresponding moment [26]. It is calculated as follows:

(6) $$F\left({x,y,t}_{p}\right)=\sum_{k=1}^{M}\sum_{i=1}^{N}W\left(x_{i}{,y}_{i}{,t}_{k}\right)\times \left[a\left(x_{i}{,y}_{i}{,\Delta t}_{k}\right)\times F\left(x_{i}{,y}_{i}{,t}_{k}\right)+b\left(x_{i}{,y}_{i}{,\Delta t}_{k}\right)\right]$$

where F(x,y,t_p_) denotes the high-resolution surface albedo of the target pixel (x,y) on the prediction date, t_p_; M denotes the number of reference dates; N denotes the total number of similar pixels (i.e., those with the same type as the target pixel) in the images; (x_i_,y_i_) denotes the position of the ith similar pixel; and a(x_i_,y_i_,Δt_k_) and b(x_i_,y_i_,Δt_k_) denote the linear fitting coefficients of the set of similar pixels of the low-spatial-resolution images between the reference moment, t_k_, and the prediction moment, t_p_, respectively, where the latter two terms are calculated using the least squares method. W(x_i_,y_i_,t_k_) denotes the weight of the ith similar pixel of the high-resolution image on the reference date, t_k_, and is calculated as follows:

(7) $$W\left(x_{i}{,y}_{i}{,t}_{k}\right){=W}_{\mathrm{individual}}\left(x_{i}{,y}_{i}{,t}_{k}\right)\times W_{\mathrm{whole}}\left(x_{i}{,y}_{i}{,t}_{k}\right)$$

where W_individual_(x_i_,y_i_,t_k_) and W_whole_(x_i_,y_i_,t_k_) denote the individual and global weights, respectively, which are calculated as follows:

(8) $$W_{\mathrm{individual}}\left(x_{i}{,y}_{i}{,t}_{k}\right)=\exp(-G\times \left\|C\left(x_{i}{,y}_{i}{,t}_{k}\right)-C\left(x_{i}{,y}_{i}{,t}_{p}\right)\right\|/h^{2})$$

(9) $$W_{\mathrm{whole}}\left(x_{i}{,y}_{i}{,t}_{k}\right)=\frac{1/\sum_{i=1}^{\omega }\sum_{j=1}^{\omega }\left(\left|C\left(x_{i}{,y}_{i}{,t}_{k}\right)-C\left(x_{i}{,y}_{i}{,t}_{P}\right)\right|\right)}{\sum_{k}\left(1/\sum_{i=1}^{\omega }\sum_{j=1}^{\omega }\left(\left|C\left(x_{i}{,y}_{i}{,t}_{k}\right)-C\left(x_{i}{,y}_{i}{,t}_{P}\right)\right|\right)\right)}$$

where h denotes the filter parameter (set to 0.15); G denotes the Gaussian kernel function; C(x_i_,y_i_,t_k_) and C(x_i_,y_i_,t_p_) denote the patches centered on the (x_i_,y_i_) pixel on the low-resolution images at the moments t_k_ and t_p_, respectively; and ω denotes the global weight with ω × ω as the window unit (set to 51).

## 3. Results

### 3.1. Accuracy Verification for STNLFFM-Based and C + STNLFFM-Based Spatiotemporally Fused Surface Algorithms

**Figure 2:**
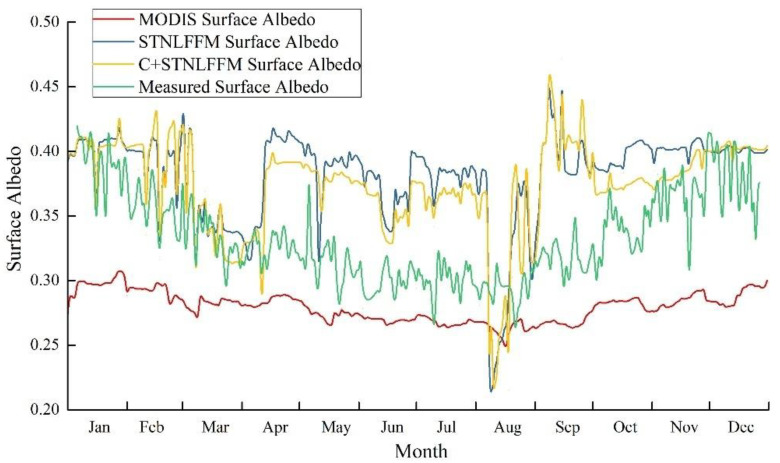
Surface albedo fusion results based on STNLFFM and C + STNLFFM at the Guaizi Lake meteorological station in 2016.

We compared the fused surface albedo corrected by the C correction model, uncorrected fused surface albedo, and MODIS-based surface albedo with the surface albedo measured at the Guaizi Lake meteorological station (Figure 2). The MODIS-based surface albedo was universally lower than the measured surface albedo; its interannual variations were consistent with those of the measured surface albedo, but its response was not sensitive to short-term variations in the measured surface albedo. The STNLFFM-based fused surface albedo, meanwhile, effectively overcame the short-term lack of sensitivity in the MODIS-based surface albedo, consistent with the observed short-term variations. However, the fused surface albedo was higher than the measured surface albedo from May to October, particularly in the summer. The C-corrected fused surface albedo retained the time-varying characteristics of the fused surface albedo, and better approximated the observational data. In addition, this approach partly alleviated the issue of the fused surface albedo being higher than the measured surface albedo.

Table 2 shows the relative errors of inter-monthly MODIS-based, STNLFFM-based fused, and C + STNLFFM-based fused surface albedo in 2016, in contrast with the measured surface albedo. The absolute values of the average relative errors of these approaches, compared with the measured surface albedo, were 16.03, 14.90, and 12.56% respectively. The STNLFFM-based fused surface albedo better approximated the observational measurements than the MODIS-based surface albedo. In January, February, November, and December, the inversion accuracy of the STNLFFM-based fused surface albedo was 84.58, 40.86, 57.83, and 79.27% higher than that of the MODIS-based surface albedo, respectively. This showed that the STNLFFM-based spatiotemporally fused surface albedo effectively improved the poor inversion accuracy of the MODIS-based surface albedo in winter. The C-corrected STNLFFM-based fused surface albedo showed improved simulation accuracy in all months except for September and December.

### 3.2. Differences in Spatiotemporal Distribution between STNLFFM-Based and C + STNLFFM-Based Fused Surface Albedo

**Figure 3:**
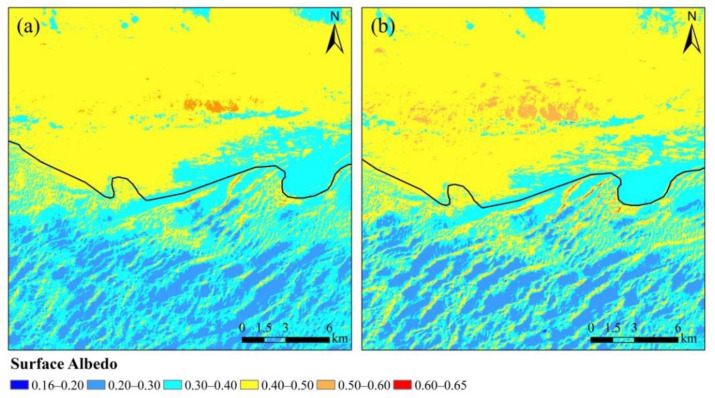
The spatial distribution differences in surface albedo based on STNLFFM (**a**) and C + STNLFFM (**b**).

Figure 3 shows the annual spatial distributions of the STNLFFM-based and C + STNLFFM-based fused surface albedo at the northern edge of the Badain Jaran Desert. There was significant spatial variation in surface albedo; specifically, the annual surface albedo outside the boundary was significantly higher than that inside the boundary. The surface albedo values over the shifting dunes and semi-shifting dunes inside the boundary mainly fell within the range of 0.2 to 0.4, the surface albedo of fixed dunes and saline-alkali land at the outer edge of the desert fell within the range of 0.4 to 0.6, and the annual surface albedo of the Gobi was mainly fell within the range of 0.4 to 0.5. The C + STNLFFM-based fused surface albedo showed more significant spatial variation, with its range expanding from 0.18–0.60 to 0.16–0.65. In addition, the spatial variation of surface albedo over both shifting dunes and semi-shifting dunes increased significantly within the desert, with the surface albedo ranges of semi-shifting dunes and shifting dunes ranging from 0.2 to 0.3 and 0.3 to 0.5, respectively. At the junction of the fixed dunes and saline-alkali land to the north of the Guaizi Lake meteorological station, the spatial variation within the STNLFFM-based fused surface albedo was relatively slight; the C + STNLFFM-based fused surface albedo was more effective at distinguishing the differences in annual average surface albedo between these two underlying surface types. Specifically, the surface albedo of the former ranged from 0.5 to 0.6, while that of the latter ranged from 0.4 to 0.5.

Table 3 displays the coefficients of correlation and variation between the monthly STNLFFM-based and C + STNLFFM-based fused surface albedo values for shifting dunes, semi-shifting dunes, fixed dunes, the Gobi, and saline-alkali land. The correlation between the STNLFFM-based and C + STNLFFM-based fused surface albedo was significant (*p* < 0.01) across different underlying surfaces in different months. This indicated that the C-corrected surface albedo effectively retained the spatiotemporal distribution characteristics of the original surface albedo. The topographic relief of the northern edge of the Badain Jaran Desert is very significant. The correlation between the STNLFFM-based and C + STNLFFM-based surface albedo was significantly lower for shifting and semi-shifting dunes than for fixed dunes, the Gobi, and saline-alkali land outside the northern edge of the Badain Jaran Desert (where the topography is relatively flat). Other than for fixed dunes in March, the coefficients of correlation between the STNLFFM-based and C + STNLFFM-based surface albedo values in the Gobi and saline-alkali land all exceeded 0.6. The coefficients of correlation between the STNLFFM-based and C + STNLFFM-based surface albedo for shifting dunes and semi-shifting dunes varied significantly across different months, while those between the STNLFFM-based and C + STNLFFM-based surface albedo in winter were higher than those in other seasons. The minimum coefficients of correlation between the STNLFFM-based and C + STNLFFM-based surface albedo always occurred in March, across all underlying surfaces. The maximum coefficients of correlation between the STNLFFM-based and C + STNLFFM-based surface albedo for fixed dunes, the Gobi, and saline-alkali land all occurred in October, while those for shifting dunes and semi-shifting dunes occurred in September. The maximum coefficients of variation of surface albedo for shifting dunes and semi-shifting dunes occurred in December (>0.2); the minimum coefficients occurred in April. Compared with shifting dunes and semi-shifting dunes, the coefficients of variation of surface albedo for fixed dunes, the Gobi, and saline-alkali land were relatively low, as were the spatial variations in their surface albedo values. Moreover, their maximum coefficients of variation all occurred in August. The minimum values for fixed dunes and saline-alkali land occurred in March, and the minimum coefficients of variation for the Gobi occurred in April. The coefficients of variation for the C + STNLFFM-based fused surface albedo were always higher than those of the STNLFFM-based fused surface albedo across different underlying surfaces. except for in February and March. This indicated that the C + STNLFFM-based surface albedo achieved enhanced spatial variation over different underlying surfaces.

## 4. Discussion

**Figure 4:**
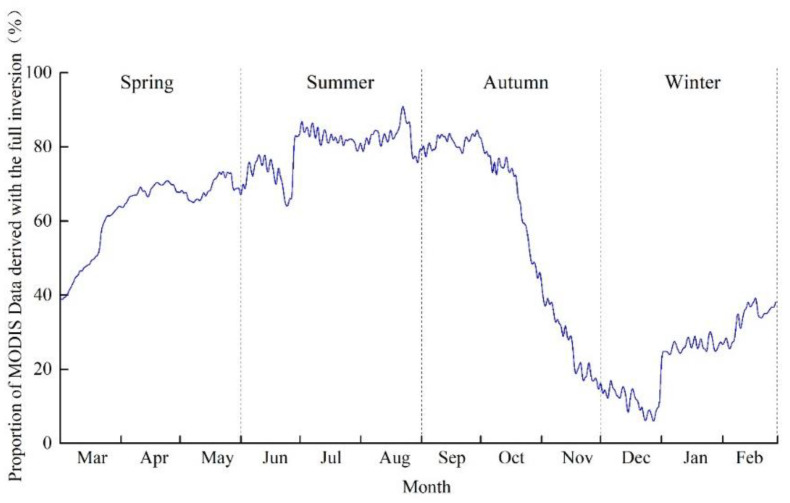
Proportion of MODIS Data derived with the full inversion over different seasons in 2016.

MODIS calculates daily surface albedo by accumulating observational data from eight days before and after the target day, and assigning different weights according to their distance from the observation date. Therefore, its sensitivity to slight, short-term changes in measured surface albedo is relatively poor [11]. The meteorological station, in contrast, mainly acquires surface albedo data according to the ratio of uplink to downlink shortwave radiation fluxes, as measured by an albedometer. This method is mainly applicable to spatial scales ranging from a few meters to tens of meters. Thus, large errors will arise when using such surface albedo data to verify the MODIS data, which has a spatial resolution of 500 m [12]. Figure 4 shows the proportion of the full inversion results of pixels in the MODIS-based surface albedo data across the whole Badain Jaran Desert in 2016. Evidently, the full inversion results in summer accounted for the highest proportion (79.55%), followed by those in spring and autumn; those in winter accounted for the lowest proportion (23.48%). There is a significant difference in elevation across the Badain Jaran Desert. Moreover, snow accumulates in high-latitude areas in winter, resulting in significant spatial variation in surface albedo. The snow albedo determined by the MODIS data inversion contains large errors because it incorporates various pixels with mixed surface types [37]. This is the main reason for the large relative errors that were observed between the simulated and measured values in January, November, and December. This indicates that in winter, the MODIS-based surface albedo data cannot meet the accuracy requirements of the climate and land surface model over the Badain Jaran Desert. In summer, the surface albedo based on OLI data inversion exhibited poor accuracy due to the influence of cloudy and rainy weather. This resulted in the poor simulation of the STNLFFM-based spatiotemporally fused surface albedo in summer. In winter, the simulation of MODIS-based surface albedo was significantly improved based on the STNLFFM, while that of the C-corrected model was used to further overcome or alleviate variations in image brightness caused by large differences in elevation. Moreover, the latter simulation may reduce the influence of accumulated snow and improve the inversion accuracy of the STNLFFM-based spatiotemporally fused surface albedo. This paper only focuses on one meteorological station. In the future, we will add more measured surface albedo from different meteorological stations inside the Badain Jaran Desert to verify C + STNLFFM accuracy, and improve the model applicability. Moreover, improving the algorithm and using high-resolution data are also necessary to obtain more accurate results.

Related studies have shown that precipitation is an important meteorological factor regarding inter-month variations in surface albedo [38]. In winter the surface albedo in snow-covered areas is significantly higher than that in uncovered areas, and there is significant spatial variation in surface albedo between different underlying surfaces. Here, the C-corrected model better captured this increased spatial variation in surface albedo between different underlying surfaces. In summer and autumn, precipitation is relatively high in most regions of China, surface vegetation cover is high, and soil moisture is high. This results in the minimum surface albedo occurring in summer. However, the mechanism that underlies the effects of vegetation cover and soil moisture on albedo remains unclear [39]. Dusty weather is frequent in spring, and the frequency of windy weather (wind speed ≥8 m·s^−1^) starts to increase in March. This dust mainly comes from the shifting dunes, with these sand grains containing a large number of high-transparency quartz particles [40]. Thus, the surface albedo is high. In addition, this extensively distributed dust increases the concentration and thickness of regional aerosols [41], thereby reducing spatial variations in surface albedo between different underlying surfaces. In such cases, the C + STNLFFM-based fused approach will over-correct surface albedo, thus reducing its inversion accuracy, and its correlation with other approaches. The shifting dunes at the northern edge of the desert are mainly distributed contiguously, with a low degree of separation; this area is dominated by rudimentary dunes (e.g., small crescent-shaped dunes and low longitudinal dunes). Its morphological structure is unstable, as it is prone to influence from dusty weather [42]. Areas where the semi-shifting dunes are interlaced with shifting dunes are only contiguous at the edge of the desert. These areas have an unstable structure and are susceptible to the external environment. The fixed dunes, the Gobi, and saline-alkali land have slight topographic reliefs at the periphery of the desert. These areas have a more stable morphological structure that is not prone to change, which explains why the coefficients of variation for the monthly surface albedo measurements in these areas were significantly lower than those observed over shifting dunes and semi-shifting dunes. The coefficients of variation of surface albedo did not change significantly after being corrected by the C-correction model.

Table 4 lists the q-values of months, underlying surfaces, and the DEM with respect to STNLFFM-based and C + STNLFFM-based spatiotemporally fused surface albedo, as calculated by GeoDetector. The q-values of months, types of underlying surfaces, and DEM all tended to increase with respect to the C + STNLFFM-based fused surface albedo; they were 27.82%, 11.8%, and 60.93% higher than those of the STNLFFM-based fused surface albedo, respectively. This indicated that the explanatory power of months and types of underlying surfaces on surface albedo increased. Namely, the degree of differentiation of surface albedo increased between different months and between different types of underlying surfaces, and the spatial variation of surface albedo was enhanced [43,44,45]. Table 5 lists the results of risk detection for surface albedo for different types of underlying surfaces. The spatial variation of the STNLFFM-based fused surface albedo did not differ significantly between shifting dunes and semi-shifting dunes, between fixed dunes and saline-alkali land, or between the Gobi and saline-alkali land. However, the C + STNLFFM-based fused surface albedo differed significantly between different types of underlying surfaces. In addition, the average C + STNLFFM-based surface albedo values of shifting dunes, semi-shifting dunes, and saline-alkali land all tended to decline. Those of fixed dunes and the Gobi increased, and the difference in surface albedo between fixed dunes and saline-alkali land tended to increase. Therefore, the C-corrected model effectively increased the spatial variation in surface albedo between different underlying surfaces.

## 5. Conclusions

The C + STNLFFM-based fused surface albedo was 2.34% and 3.57% more accurate than the STNLFFM-based fused and MODIS-based surface albedo, respectively. In addition, it effectively responded to short-term changes in the measured surface albedo, thus overcoming the limitations of the STNLFFM-based fused surface albedo (specifically, changes in image brightness due to great differences in elevation) and MODIS-based surface albedo (specifically, poor applicability in winter). However, its accuracy was poor in summer due to the influence of cloud and rain on the quality of the OLI data.

The C + STNLFFM-based fused surface albedo exhibited enhanced variation between different underlying surfaces. Specifically, the average surface albedo tended to decrease over shifting and semi-shifting dunes and saline-alkali land, while increasing over fixed dunes and the Gobi. In addition, the spatial variation in surface albedo differed significantly between shifting dunes and semi- shifting dunes, between fixed dunes and saline-alkali land, and between the Gobi and saline-alkali land.

The developed C + STNLFFM-based fused surface albedo can effectively retain the spatiotemporal distribution characteristics of the STNLFFM-based fused surface albedo. In March, the frequent high winds and extensively distributed dust increase the concentration and thickness of regional aerosols, thereby reducing the spatial variation in surface albedo between different underlying surfaces. In this case, the C-corrected model over-corrected surface albedo, resulting in poor correlations.

## Figures and Tables

**Table 1:** OLI images obtained from orbits 132/031 and 133/031.

Path/Row	Scan Time						
132/31	12 January 2016	28 January 2016	13 February 2016	29 February 2016	16 March 2016	1 April 2016	17 April 2016
	3 May 2016	4 June 2016	20 June 2016	6 July 2016	22 July 2016	7 August 2016	23 August 2016
	8 September 2016	24 September 2016	10 October 2016	27 November 2016	13 December 2016		
133/31	20 February 2016	8 April 2016	24 April 2016	26 May 2016	11 June 2016	27 June 2016	13 July 2016
	29 July 2016	30 August 2016	15 September 2016	1 October 2016	17 October 2016	02 November 2016	

**Table 2:** The inter-monthly RE between measured values and MODIS surface albedo, STNLFFM surface albedo, and C + STNLFFM surface albedo (%).

Month	MODIS—MV	STNLFFM—MV	C + STNLFFM—MV
January 2016	−23.47	3.62	3.30
February 2016	−17.45	10.32	12.56
March 2016	−15.69	5.80	3.29
April 2016	−12.78	16.59	11.84
May 2016	−12.87	23.62	20.82
June 2016	−10.81	21.73	16.88
July 2016	−10.88	27.74	22.15
August 2016	−9.36	12.19	11.03
September 2016	−15.83	24.97	27.39
October 2016	−15.98	17.63	11.31
November 2016	−22.65	9.55	4.65
December 2016	−24.58	5.10	5.44

RE and MV represent Relative Error and Measured Value, respectively.

**Table 3:** The inter-monthly correlation coefficients between STNLFFM surface albedo and C + STNLFFM surface albedo over shifting dunes, semi-shifting dunes, fixed dunes, the Gobi, and saline-alkali land, and the surface albedo coefficients of variation of shifting dunes, semi-shifting dunes, fixed dunes, the Gobi, and saline-alkali land based on STNLFFM and C + STNLFFM.

Month	Shifting Dunes			Semi-Shifting Dunes			Fixed Dunes			Gobi			Saline-Alkali Land		
	Correlation Coefficient	STNLFFM CV	C + STNLFFM CV	Correlation Coefficient	STNLFFM CV	C + STNLFFM CV	Correlation Coefficient	STNLFFM CV	C + STNLFFM CV	Correlation Coefficient	STNLFFM CV	C + STNLFFM CV	Correlation Coefficient	STNLFFM CV	C + STNLFFM CV
January 2016	0.806 **	0.2352	0.2494	0.864 **	0.2336	0.2606	0.895 **	0.1236	0.1294	0.934 **	0.0693	0.0727	0.860 **	0.0834	0.0869
February 2016	0.743 **	0.2075	0.1893	0.771 **	0.2034	0.1820	0.832 **	0.0983	0.0911	0.888 **	0.0659	0.0643	0.829 **	0.0746	0.0671
March 2016	0.502 **	0.1430	0.1215	0.323 **	0.1442	0.1225	0.333 **	0.0543	0.0671	0.626 **	0.0681	0.0694	0.651 **	0.0522	0.0561
April 2016	0.703 **	0.1251	0.1448	0.592 **	0.1136	0.1625	0.764 **	0.0708	0.1009	0.851 **	0.0495	0.0565	0.759 **	0.0621	0.0684
May 2016	0.708 **	0.1351	0.1496	0.632 **	0.1315	0.1799	0.613 **	0.0748	0.0949	0.804 **	0.0588	0.0652	0.702 **	0.0528	0.0669
June 2016	0.739 **	0.1377	0.1514	0.690 **	0.1404	0.2002	0.809 **	0.1038	0.1518	0.880 **	0.0694	0.0757	0.666 **	0.0686	0.0816
July 2016	0.731 **	0.1904	0.1903	0.730 **	0.2190	0.2416	0.907 **	0.1038	0.1357	0.919 **	0.0629	0.0678	0.812 **	0.0687	0.0771
August 2016	0.669 **	0.1582	0.1630	0.556 **	0.1497	0.1853	0.790 **	0.1808	0.2162	0.744 **	0.1114	0.1321	0.772 **	0.1526	0.1559
September 2016	0.904 **	0.1697	0.1781	0.863 **	0.1341	0.1787	0.841 **	0.0960	0.1092	0.778 **	0.0661	0.0745	0.846 **	0.0738	0.0833
October 2016	0.851 **	0.1527	0.2020	0.854 **	0.1577	0.2336	0.927 **	0.0993	0.1253	0.926 **	0.0605	0.0659	0.899 **	0.0645	0.0803
November 2016	0.574 **	0.1858	0.2521	0.526 **	0.2038	0.2732	0.713 **	0.0927	0.1279	0.856 **	0.0671	0.0707	0.739 **	0.0681	0.0885
December 2016	0.807 **	0.2600	0.2641	0.861 **	0.2581	0.2705	0.882 **	0.1216	0.1217	0.938 **	0.0696	0.0699	0.836 **	0.0853	0.0872

** *p* < 0.01; CV represents coefficients of variation.

**Table 4:** PD values of month, underlying surfaces, and DEM for surface albedo based on STNLFFM and C + STNLFFM.

	Month	Underlying Surfaces	DEM
STNLFFM	0.0346 **	0.4039 **	0.0434 **
C + STNLFFM	0.0442 **	0.4519 **	0.0698 **

** *p* < 0.01.

**Table 5:** Risk detector of surface albedo over different underlying surfaces.

	Shifting Dunes	Semi-Shifting Dunes	Fixed Dunes	Gobi	Saline-Alkali Land
Shifting dunes					
Semi-shifting dunes	N				
Fixed dunes	Y	Y			
Gobi	Y	Y	Y		
saline-alkali land	Y	Y	N	N	
Mean of STNLFFM surface albedo	0.3657	0.3073	0.4334	0.4573	0.4207
Shifting dunes					
Semi-shifting dunes	Y				
Fixed dunes	Y	Y			
Gobi	Y	Y	Y		
saline-alkali land	Y	Y	Y	Y	
Mean of C + STNLFFM surface albedo	0.3507	0.2961	0.5214	0.4453	0.3934

N and Y represent No significant difference and Significant difference, respectively.
